# Grain refinement of commercially pure aluminum with addition of Ti and Zr elements based on crystallography orientation

**DOI:** 10.1038/s41598-020-73799-2

**Published:** 2020-10-06

**Authors:** Zhongwei Chen, Kang Yan

**Affiliations:** State Key Laboratory of Solidification Processing, Northwestern Polytechnical University, Xi’an, 710072 China

**Keywords:** Metals and alloys, Characterization and analytical techniques

## Abstract

The edge-to-edge matching (E2EM) model and electron back-scatter diffraction (EBSD) technique are used to explore the grain refinement mechanism of commercially pure Al through the addition of Ti and Zr elements. EBSD results show that there are favorable crystallography orientation relationships (ORs) between both Al_3_Ti and Al_3_Zr particles with α-Al matrix. Due to these ORs Al_3_Ti and Al_3_Zr particles act as the heterogeneous nucleation site during solidification nucleation of Al–Ti and Al–Zr alloys, respectively. Furthermore, both Al_3_Ti and Al_3_Zr particles have small values of interplanar spacing mismatch and interatomic spacing misfit with respect to α-Al matrix by using E2EM. It shows that micro-addition of Ti and Zr element is efficient heterogeneous nucleation refiner in commercial purity Al or Al alloys. Besides, there may be some other mechanisms in grain refinement of Al alloys with addition of Ti grain refiner.

## Introduction

The grain refinement of commercial purity aluminum and its alloys by adding inoculant to get uniform, fine microstructure in casting progress is a common practice in commercial production which not only improves mechanical properties but also aids in subsequent deformation processes^1–3^. The processes of inoculation improves the quality of wrought alloy by eliminating the columnar structure. A lot of research has already been done to work out the efficient grain refiners and theories to support the mechanism of grain refinement^4–9^. However, there is no such theory that can comprehend the phenomenon of grain refinement completely except the concepts of numerous potent nucleants and sufficient effective solutes. According to nucleation theory, numerous potent nucleants facilitates α-Al nucleation at small undercooling while sufficient effective solutes provide undercooling which promotes the nucleation of adjacent nucleant particles during solidification of Al alloys^10–12^. Despite all the existing theories, there are still a lot to be answered. What are the crucial factors during the grain refinement are yet to be determined. Considering the existing theories, potency of nucleate in grain refinement can be divided into two groups. One hypothesis is that an efficient nucleate should minimize the interfacial energy between matrix and nuclei through coherent crystallographic orientation. The Turnbull and Vonnegut model, the Bramfitt model and the edge-to-edge matching (E2EM) model are the modified forms of this theory^13–15^. In another model, free growth model, emphasizes on the size of nucleant particles and proposes that an efficient nucleant particle should be larger than a critical threshold value so that the nucleation can progress effectively^16^. However, the real and explicit grain refinement mechanism in cast metals and alloys is still not fully comprehended and there exist a lot of controversies.

A large amount of literature studies the grain refinement of cast aluminum alloys, which are roughly divided into physical methods and chemical methods. The physical method refines the grain by introducing physical factors into the solidification process. After using ultrasonic treatment in the solidification process, the grain size of commercial pure aluminum and Al–Si–Cu alloy is greatly refined compared to the alloy without ultrasonic treatment^17,18^. By applying electric and magnetic fields of different strengths during the solidification process, the grains of the Al–Si alloy are also greatly refined^19^. The chemical method mainly refines the grain by adding grain refiner to the aluminum alloy. As the most common Al alloy grain refiners in industrial production, Al–Ti-B and Al–Ti-C systems had been previously investigated^3,20^. Considering the effectiveness of grain refiners, many researchers has used Ti, Zr, Nb, etc. to study their effects on grain refinement of aluminum alloys^21–23^. Till date Ti element is considered as the best inoculant for grain refinement of aluminum alloys. However, the peritectic approach contradicts this theory that adding Ti can result in sufficient grain refinement unless it is added to its maximum solubility limit in α-Al matrix which results in peritectic reaction. This phenomenon raises a question of whether a similar result can be obtained by adding other peritectic-forming solutes. Feng Wang et al. had investigated the grain refinement of Al alloys by adding Nb, Zr and V, and the significant amount of grain refinement was observed during his experiments^24,25^. They also attribute the results of grain refinement to in-situ formation of pro-peritectic particles as the solutes concentrations are above their maximum solubilities. Yang Li et al. found that when Al–Si-B is used to refine Al-Si alloys, Si poisoning will occur. Si atoms will segregate at the interface between Al_3_Ti and α-Al, making the refinement efficiency of Al–Ti and Al–Ti-B master alloy weaker^26,27^. Unfortunately, there is no exactly interpretation about how Ti element is a better grain refining agent for Al alloys when compared with other peritectic-forming solutes.

It is well known that a low interfacial energy between the nucleant particle and the matrix in solidification progress has a great significance in improving the potency of the nucleant particles^3,20^. In general, a good crystallography matching between the nucleant particle and the matrix promotes a low interfacial energy, which favors the formation of uniform and fine cast structure^13^. Crystallographic matching usually refers the lattice matching in conventional research, which only suits for simple crystal structure. However, the majority of the nucleant particles have a complex crystal structure, for which the crystallographic matching is more than lattice matching of the interfaces^24^.

The edge-to-edge matching model was developed by Zhang and Kelly and it was used to predict the habit plane and orientation relationship (OR) in diffusion controlled solid-state phase transformation. E2EM model was based on several critical assumptions: the interface between two phases must be coherent or semi-coherent to get low surface energy, that is, two phases should be have a good crystallography relationship with each other. Besides, the minimum interfacial energy requires low strain energy in the interface, that is, the close-packed or nearly close-packed atom rows in each phase in the interface should be parallel with each other, and these matching rows can be either straight or zigzag rows and straight rows matching with straight rows and zigzag rows with zigzag rows^28,29^. The matching degree of rows and matching planes are evaluated with interatomic spacing misfit (*f*_r_) and interplannar spacing misfit (*f*_d_). Small *f*_r_ and *f*_d_ refers to good matching at the interface, while *f*_r_ and *f*_d_ are defined as follows:

1 $$ f_{{\text{r}}} = \frac{{\left| {r_{{\text{M}}} - r_{{\text{P}}} } \right|}}{{r_{{\text{P}}} }}\;{\text{and }}f_{{\text{d}}} = \frac{{\left| {d_{{\text{M}}} - d_{{\text{P}}} } \right|}}{{d_{{\text{P}}} }} $$

rather than

2 $$ f_{{\text{r}}} = \frac{{\left| {r_{{\text{M}}} - r_{{\text{P}}} } \right|}}{{r_{{\text{M}}} }}\;{\text{and }}f_{{\text{d}}} = \frac{{\left| {d_{{\text{M}}} - d_{{\text{P}}} } \right|}}{{d_{{\text{M}}} }} $$

where *r*_M_ is the interatomic spacing of matching row of metal matrix while *r*_P_ is the interatomic spacing of matching row of inoculant particle, and *d*_M_ is the interplanar spacing of matching row of metal matrix while *d*_P_ is the interplanar spacing of matching row of inoculant particle. A efficient inoculant should have low *f*_r_ and *f*_d_ with metal matrix, in general, *f*_r_ and *f*_d_ should be lower than 10%^30,31^.

Electron Back Scattering Diffraction (EBSD) is a technique based on the analysis of the Kikuchi pattern by the excitation of the electron beam on the surface of the sample. EBSD has a unique advantage in the determination of the crystal orientation and microstructure compared with the traditional analysis methods. It is used to study the grain boundary, its types, misorientation and its distribution statically as well quantitively. EBSD has emerged as a one of the strongest characterization tools that provides valuable information about the mechanisms of nucleation and growth during modification, and agglomeration of equiaxed crystals in secondary solidification^32–34^.

Several scientific metallurgical tools has been used to study and understand the mechanism of grain refinement using E2EM model, however EBSD has rarely been used to study the crystallography orientation relations (ORs) between nucleant particle and matrix which is important to the predicted result from E2EM model^28,29^. In this work, EBSD technique is used to characterize the crystallography ORs both Al_3_Zr/Al and Al_3_Zr/Al in commercially pure aluminum with addition of Ti and Zr elements, and the calculating results obtained by using E2EM model account for experimental results correspondingly, in which grain refinement mechanism of aluminum and Al alloys can be clarified based on crystallography orientation.

## Methods

Commercial purity aluminum (99.995%) was used to study the grain refinement in this work. Pure aluminum was first melted at 720 °C in a resistance furnace and then grain refined by addition of Al-5 wt.%Ti and Al-4 wt.%Zr master alloys. Four melts of Al-0.1 wt.%Ti, Al-0.2 wt.%Ti, Al-0.1 wt.%Zr and Al-0.2 wt.%Zr alloys were casted in metal mould which was preheated at 200 °C. In order to obtain uniform casting, the specimens, size 50 mm in diameter and 100 mm length, were cut from the middle of the ingots.

**Figure 1:**
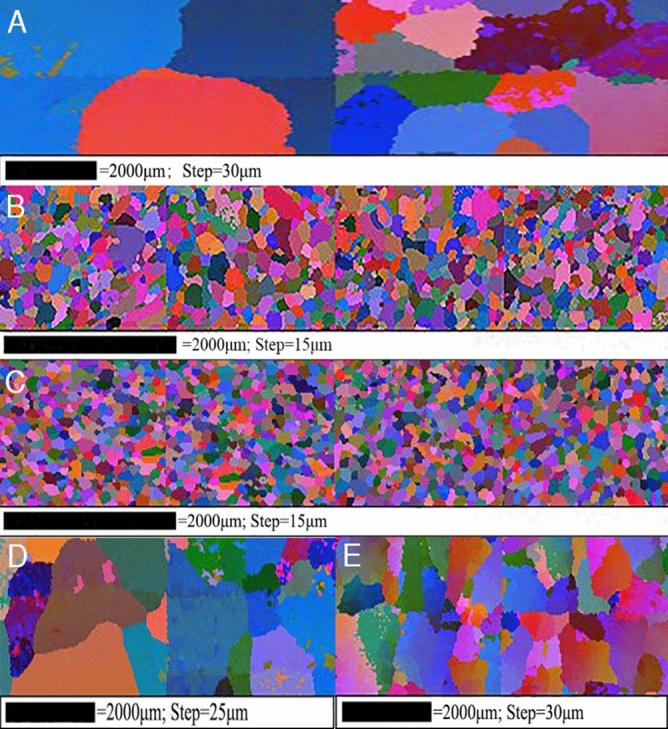
EBSD grain reconstruction mappings of Al and its alloys in free solidification: (**A**) purity Al (**B**) Al-0.1 wt.%Ti (**C**) Al-0.2 wt.%Ti (**D**) Al-0.1 wt.%Zr (**E**) Al-0.2 wt.%Zr.

**Figure 2:**
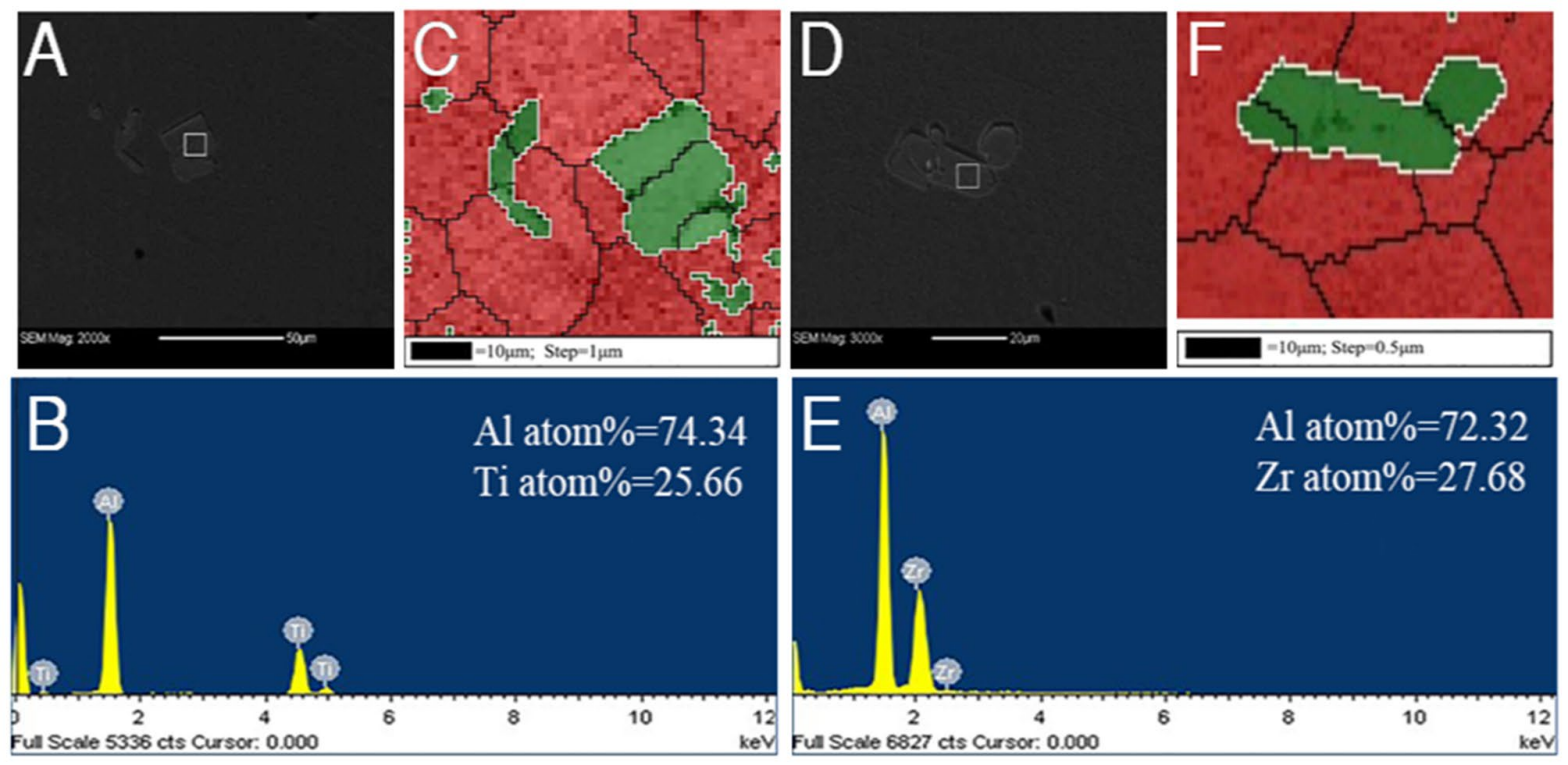
Microstructure and EDS in free solidification: (**A**) SEM of Al-0.2 wt.%Ti alloy (**B**) corresponding EDS spectrogram of Al-0.2 wt.%Ti alloy, (**C**) EBSD phase distribution mapping of Al-0.2 wt.%Ti alloy, (**D**) SEM of Al-0.2 wt.%Zr alloy (**E**) corresponding EDS spectrogram of Al-0.2 wt.%Zr alloy, (**F**) EBSD phase distribution mapping of Al-0.2 wt.%Zr alloy.

For EBSD experiments, the samples were first polished mechanically as per standard procedures. In order to remove the surface deformation layer, electron polishing at 30 V for 5 s ~ 15 s was chosen with the standard A2-struers solution containing 144 ml ethanol, 40 ml 2-butoxyethanol and 16 ml perchloric acid. Software Channel 5 of HKL technology, which is installed in field emission gun scanning electron microscope (FEG-SEM, ZEISS-SUPRA55), was used for EBSD examinations. The acceleration voltage in EBSD test is 20 kV, the step size parameter is in the legend of each grain reconstruction diagram in Figs. 1 and 2. Tango-Maps in the channel 5 was used for grain reconstruction and subset selection, and Mambo-Pole figures in the channel 5 was used for pole figure acquisition and processing. An energy dispersive spectroscope (EDS-OXFORD) attached in FEG-SEM was utilized to determine the chemical compositions of the nucleant particles. The acceleration voltage in EDS test is 20 kV.

## Results and discussion

### Grain refinement

Figure 1 shows the EBSD grain reconstruction mappings of as-cast commercial purity aluminum (Fig. 1A) with different content additions of Ti element (Fig. 1B,C) and Zr (Fig. 1D,E) element. It is obvious that the large columnar grains of microstructure with the addition of 0.1 wt.%Zr (Fig. 1D) is similar to that of pure aluminum. Nevertheless, with the addition of 0.2 wt.%Zr (Fig. 1E) or 0.1 wt.% ~ 0.2 wt.%Ti (Fig. 1B,C), both clear transition from columnar to equiaxed grains and significant grain refinement in microstructure are observed. It is observed that the average grain size is decreased from 1500 to 1200 μm with addition of 0.1 wt.%Zr (Fig. 1D), while with addition of 0.2 wt.%Zr (Fig. 1E), the grain size is significantly decreased to approximately 400 μm in which the amount of Zr addition exceeds the maximum solubility (0.11 wt.%Zr). With the addition of 0.1 wt.%Ti (Fig. 1B) and 0.2 wt.%Ti (Fig. 1C), the average grain size is remarkably reduced to 100 μm and 80 μm. Results indicate that the grain refinement efficiency of Ti is more significant than that of Zr when equal amount of Ti and Zr is added to pure aluminum.

### ORs from EBSD results

The samples with addition 0.2 wt.%Zr and 0.2 wt.%Ti were examined by SEM to reveal the detailed microstructure. Figure 2 shows SEM microstructure of Al-0.2 wt.%Ti and Al-0.2 wt.%Zr alloy in free solidification and corresponding EDS spectrogram and EBSD phase distribution mappings. It is found that there are small particles reproducibly in the refined grains, as shown in Fig. 2A,D. These particles are deemed to serve as heterogeneous nucleation sites during solidification. Figure 2B,E give typical EDS spectrums of the particles observed at or near the grain center. EDS analyses indicate that the composition of these particles are abundant in Al and Ti or Zr, in which the approximate atomic ratio of Al and Ti or Zr is closely 3:1. Combining EDS results and the equilibrium phase diagrams of binary Al-Ti and Al-Zr, it is sure that those particles observed at or near the grain center in Al-Ti alloy and Al-Zr alloy are Al_3_Ti particles and Al_3_Zr particles, respectively. This further demonstrates the hypothesis that Al_3_Ti or Al_3_Zr particles at grain center are possibly the nucleants, which are responsible for grain refinement obtained with addition of Ti or Zr element in pure aluminum.

According to nucleation theory and E2EM model, an efficient heterogeneous nucleation core particle must have a good crystallographic orientation matching with its matrix phase. It is necessary for matrix atom to attach itself with heterogeneous nucleation core particle. Therefore, it is imperative to study the crystallographic orientation relationship between α-Al matrix and heterogeneous nucleation core particles, including Al_3_Ti and Al_3_Zr particles.

**Figure 3:**
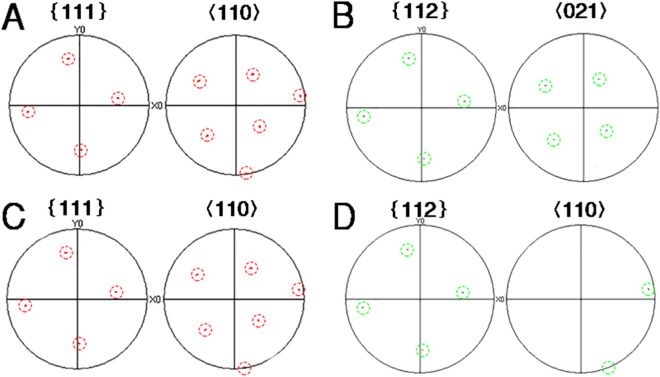
Al-0.2 wt.%Ti alloy in free solidification: (**A**,**C**) Pole figures of α-Al (**B**,**D**) Pole figures of Al_3_Ti particles (The red dotted circles represent pole dots of α-Al, and the green represent pole dots of Al_3_Ti particles).

To experimentally verify the claims, the ORs between Al_3_Ti particles near grain center and α-Al matrix were determined using automated EBSD. Figure 2C shows typical EBSD phase distribution mappings. Pole figures of Al phase and Al_3_Ti particles are shown in Fig. 3. Figure 3A,C are pole figures of Al phase, while Fig. 3B,D are the pole figures of Al_3_Ti particles. Figure 3A,B represent a kind of crystallography relationship between Al and Al_3_Ti, Fig. 3C,D represent the other. Although the pole dots of Al_3_Ti particles are less than those of Al phase due to lesser Al_3_Ti particles, the pole figures can characterize their ORs. Pole figure results shows that there are two kinds of crystallography orientation between Al phase and Al_3_Ti particles. That is,

$$ {\text{OR}}(1):\left\{ {111} \right\}_{Al} //\left\{ {112} \right\}_{{Al_{3} Ti}} ,\left\langle {110} \right\rangle_{Al} //\left\langle {110} \right\rangle_{{Al_{3} Ti}} $$

$$ {\text{OR}}(2):\left\{ {111} \right\}_{Al} //\left\{ {112} \right\}_{{Al_{3} Ti}} ,\left\langle {110} \right\rangle_{Al} //\left\langle {021} \right\rangle_{{Al_{3} Ti}} $$

**Figure 4:**
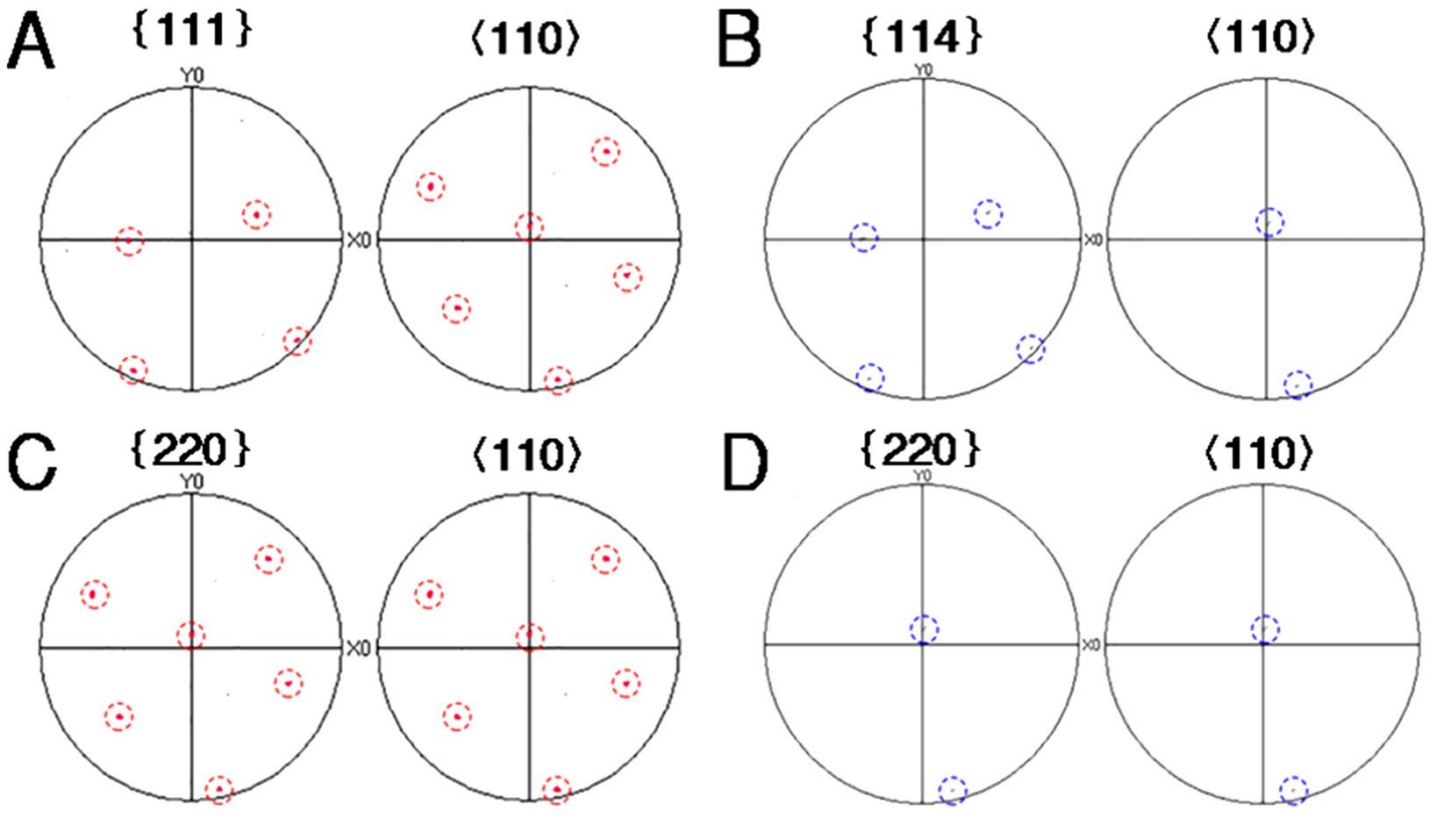
Al-0.2 wt.%Zr alloy in free solidification: (**A**,**C**) Pole figures of α-Al (**B**,**D**) Pole figures of Al_3_Zr particles (The red dotted circles represent pole dots of α-Al, and the blue represent pole dots of Al_3_Zr particles).

Figure 4 describes that pole figures of Al phase and Al_3_Zr particles from EBSD phase distribution mappings in Fig. 2F. Figure 4A,C are the pole figures of Al phase, while Fig. 4B,D are the pole figures of Al_3_Zr particles. Therefore, ORs shown in Fig. 4 are expressed as:

$$ {\text{OR}}(1):\left\{ {111} \right\}_{Al} //\left\{ {114} \right\}_{{Al_{3} Zr}} ,\left\langle {110} \right\rangle_{Al} //\left\langle {110} \right\rangle_{{Al_{3} Zr}} $$

$$ {\text{OR}}(2):\left\{ {220} \right\}_{Al} //\left\{ {220} \right\}_{{Al_{3} Zr}} ,\left\langle {110} \right\rangle_{Al} //\left\langle {110} \right\rangle_{{Al_{3} Zr}} $$

The summary of results for the crystallography orientation relationship between Al/Al_3_Ti and Al/Al_3_Zr during these experiments is listed in Table 1.

**Table 1:** Crystallography orientation relationships both Al/Al_3_Ti and Al/Al_3_Zr from E2EM prediction and EBSD experiments.

ORs	EBSD experiments	E2EM prediction
Al/Al_3_Ti	${\left\{111\right\}}_{Al}//{\left\{112\right\}}_{{Al}_{3}Ti}$,${\langle 110\rangle }_{Al}//{\langle 110\rangle }_{{Al}_{3}Ti}$ ${\left\{111\right\}}_{Al}//{\left\{112\right\}}_{{Al}_{3}Ti}$,${\langle 110\rangle }_{Al}//{\langle 021\rangle }_{{Al}_{3}Ti}$	${\left\{111\right\}}_{Al}//{\left\{112\right\}}_{{Al}_{3}Ti}$,${\langle 110\rangle }_{Al}//{\langle 110\rangle }_{{Al}_{3}Ti}$, ${f}_{\rm{d}}=1.65\%$, ${f}_{\rm{r}}=5\%$ ${\left\{111\right\}}_{Al}//{\left\{112\right\}}_{{Al}_{3}Ti}$,${\langle 110\rangle }_{Al}//{\langle 021\rangle }_{{Al}_{3}Ti}$, ${f}_{\rm{d}}=1.65\%$, ${f}_{\rm{r}}=0.73\%$ ${\left\{111\right\}}_{Al}//{\left\{112\right\}}_{{Al}_{3}Ti}$,${\langle \stackrel{-}{2}11\rangle }_{Al}//{\langle 111\rangle }_{{Al}_{3}Ti}$, ${f}_{\rm{d}}=1.65\%$, ${f}_{\rm{r}}=2.46\%$ ${\left\{111\right\}}_{Al}//{\left\{004\right\}}_{{Al}_{3}Ti}$,${\langle 110\rangle }_{Al}//{\langle 110\rangle }_{{Al}_{3}Ti}$, ${f}_{\rm{d}}=8.95\%$,${f}_{\rm{r}}=5\%$
Al/Al_3_Zr	${\left\{111\right\}}_{Al}//{\left\{114\right\}}_{{Al}_{3}Zr}$,${\langle 110\rangle }_{Al}//{\langle 110\rangle }_{{Al}_{3}Zr}$ ${\left\{220\right\}}_{Al}//{\left\{220\right\}}_{{Al}_{3}Zr}$,${\langle 110\rangle }_{Al}//{\langle 110\rangle }_{{Al}_{3}Zr}$	${\left\{111\right\}}_{Al}//{\left\{114\right\}}_{{Al}_{3}Zr}$,${\langle 110\rangle }_{Al}//{\langle 110\rangle }_{{Al}_{3}Zr}$, ${f}_{\rm{d}}=1.34\%$, ${f}_{r}=1.05\%$ ${\left\{111\right\}}_{Al}//{\left\{114\right\}}_{{Al}_{3}Zr}$,${\langle 110\rangle }_{Al}//{\langle 40\stackrel{-}{1}\rangle }_{{Al}_{3}Zr}$, ${f}_{\rm{d}}=1.34\%$, ${f}_{\rm{r}}=1.99\%$ ${\left\{111\right\}}_{Al}//{\left\{114\right\}}_{{Al}_{3}Zr}$,${\langle \stackrel{-}{2}11\rangle }_{Al}//{\langle 22\stackrel{-}{1}\rangle }_{{Al}_{3}Zr}$, ${f}_{\rm{d}}=1.34\%$, ${f}_{r}=1.05\%$ ${\left\{220\right\}}_{Al}//{\left\{220\right\}}_{{Al}_{3}Zr}$,${\langle 110\rangle }_{Al}//{\langle 110\rangle }_{{Al}_{3}Zr}$, ${f}_{d}=1.05\%$, ${f}_{r}=1.05\%$ ${\left\{220\right\}}_{Al}//{\left\{220\right\}}_{{Al}_{3}Zr}$,${\langle \stackrel{-}{2}11\rangle }_{Al}//{\langle 22\stackrel{-}{1}\rangle }_{{Al}_{3}Zr}$, ${f}_{\rm{d}}=1.65\%$,${f}_{r}=1.5\%$

### ORs from E2EM model prediction

Despite the fact that an efficient heterogeneous nucleation core particle must has a good crystallographic orientation matching with its matrix phase, its mismatch with matrix phase should be small so that there can exist low strain energy in the interface of heterogeneous nucleation core particle and matrix phase. Good crystallographic orientation matching and low lattice mismatch, both of these conditions are beneficial to reduce the energy of heterogeneous nucleation and promote nucleation.

α-Al phase has a face-centred cubic (fcc) crystal structure and it has a close-packed direction $\langle 110\rangle$ and a zigzag close-packed direction $\langle \stackrel{-}{2}11\rangle$. Resultantly it has a close-packed plane $\left\{111\right\}$, which contains $\langle 110\rangle$ and $\langle \stackrel{-}{2}11\rangle$ close-packed rows, and two near close-packed planes $\left\{020\right\}$ (contains $\langle 110\rangle$ close-packed row) and $\left\{220\right\}$ (contains $\langle 110\rangle$ and $\langle \stackrel{-}{2}11\rangle$ close-packed rows). Al_3_Ti particle has a crystal structure of tetragonal crystal and its most close-packed plane is $\left\{112\right\}$, which contains three crystal orientations: $\langle 021\rangle$, $\langle 111\rangle$ and $\langle 110\rangle$. Its near close-packed plane $\left\{004\right\}$ contains one close-packed row $\langle 110\rangle$ while the other close-packed plane $\left\{020\right\}$ contains one close-packed row $\langle 021\rangle$.

On the basis of the identified close-packed planes and rows, the values of *f*_d_ and *f*_r_ between Al phase and Al_3_Ti particles were calculated. The values of *f*_r_ were calculated by coupling the same types of atomic row, that is, zigzag rows match with zigzag rows and straight rows match with straight. The ORs between Al phase and Al_3_Ti particles (for which misfit degree is less than 10% ) are: ${\langle 110\rangle }_{Al}//{\langle 110\rangle }_{{Al}_{3}Ti}$, *f*_r_ = 5%; ${\langle 110\rangle }_{Al}//{\langle 021\rangle }_{{Al}_{3}Ti}$, *f*_r_ = 0.73%; ${\langle \stackrel{-}{2}11\rangle }_{Al}//{\langle 111\rangle }_{{Al}_{3}Ti}$, *f*_r_ = 2.46%. The interplanar spacing mismatches between α-Al phase and Al_3_Ti particles are given as ${\left\{111\right\}}_{Al}//{\left\{112\right\}}_{{Al}_{3}Ti}$, *f*_d_ = 1.65%, ${\left\{111\right\}}_{Al}//{\left\{004\right\}}_{{Al}_{3}Ti}$, *f*_d_ = 8.95%. The possible crystal orientation relationships between α-Al phase and Al_3_Ti particles ,drawn from the aforementioned observation, is as following:

$$ {\text{OR}}(1):\left\{ {111} \right\}_{Al} //\left\{ {112} \right\}_{{Al_{3} Ti}} ,\left\langle {110} \right\rangle_{Al} //\left\langle {110} \right\rangle_{{Al_{3} Ti}} $$

$$ {\text{OR }}\left( {2} \right):\left\{ {111} \right\}_{Al} //\left\{ {112} \right\}_{{Al_{3} Ti}} ,\left\langle {110} \right\rangle_{Al} //\left\langle {021} \right\rangle_{{Al_{3} Ti}} $$

$$ {\text{OR }}\left( {3} \right):\left\{ {111} \right\}_{Al} //\left\{ {112} \right\}_{{Al_{3} Ti}} ,\left\langle {\overline{2}11} \right\rangle_{Al} //\left\langle {111} \right\rangle_{{Al_{3} Ti}} $$

$$ {\text{OR }}\left( {4} \right):\left\{ {111} \right\}_{Al} //\left\{ {004} \right\}_{{Al_{3} Ti}} ,\left\langle {110} \right\rangle_{Al} //\left\langle {110} \right\rangle_{{Al_{3} Ti}} $$

Al_3_Zr particle has a crystal structure of tetragonal crystal and its most close-packed plane is $\left\{114\right\}$. It contains three crystal orientations: $\left\langle {22\overline{1}} \right\rangle$, $\left\langle {110} \right\rangle$ and $\left\langle {40\overline{1}} \right\rangle$. Its near close-packed plane, $\left\{020\right\}$, contains one close-packed row $\langle 40\stackrel{-}{1}\rangle$ and its another close-packed plane $\left\{220\right\}$ contains two close-packed rows, $\langle 22\stackrel{-}{1}\rangle$ and $\langle 110\rangle$. On the basis of the identified close-packed planes and rows, the values of *f*_d_ and *f*_r_ between α-Al phase and Al_3_Zr particles were calculated. The ORs between α-Al phase and Al_3_Zr particles (for which misfit degree of value less than 10%) are: ${\langle 110\rangle }_{\rm{Al}}//{\langle 110\rangle }_{{\rm{Al}}_{3}\rm{Zr}}$, ${\langle \stackrel{-}{2}11\rangle }_{\rm{Al}}//{\langle 22\stackrel{-}{1}\rangle }_{{\rm{Al}}_{3}\rm{Zr}}$, *f*_r_ = 1.05%; ${\langle 110\rangle }_{\rm{Al}}//{\langle 40\stackrel{-}{1}\rangle }_{{\rm{Al}}_{3}\rm{Zr}}$, *f*_r_ = 1.99%. The interplanar spacing mismatches between α-Al phase and Al_3_Zr particles are ${\left\{111\right\}}_{\rm{Al}}//{\left\{114\right\}}_{{\rm{Al}}_{3}\rm{Zr}}$, *f*_d_ = 1.34%; ${\left\{220\right\}}_{\rm{Al}}//{\left\{220\right\}}_{{\rm{Al}}_{3}\rm{Zr}}$, *f*_d_ = 1.05%. Therefore, the possible crystal orientation relationships between α-Al phase and Al_3_Zr particles are:

$${\rm{OR }(1): \left\{111\right\}}_{Al}//{\left\{114\right\}}_{{Al}_{3}Zr},{\langle 110\rangle }_{Al}//{\langle 110\rangle }_{{Al}_{3}Zr}$$

$${\rm{OR }(2): \left\{111\right\}}_{Al}//{\left\{114\right\}}_{{Al}_{3}Zr},{\langle 110\rangle }_{Al}//{\langle 40\stackrel{-}{1}\rangle }_{{Al}_{3}Zr}$$

$${\rm{OR }(3): \left\{111\right\}}_{Al}//{\left\{114\right\}}_{{Al}_{3}Zr},{\langle \stackrel{-}{2}11\rangle }_{Al}//{\langle 22\stackrel{-}{1}\rangle }_{{Al}_{3}Zr}$$

$${\rm{OR }(4): \left\{220\right\}}_{Al}//{\left\{220\right\}}_{{Al}_{3}Zr},{\langle 110\rangle }_{Al}//{\langle 110\rangle }_{{Al}_{3}Zr}$$

$${\rm{OR }(5): \left\{220\right\}}_{Al}//{\left\{220\right\}}_{{Al}_{3}Zr},{\langle \stackrel{-}{2}11\rangle }_{Al}//{\langle 22\stackrel{-}{1}\rangle }_{{Al}_{3}Zr}$$

All ORs results both Al/Al_3_Ti and Al/Al_3_Zr were listed Table 1. Although ORs results from E2EM prediction are more than those from EBSD experiments, which is due to the limitations of the experiment, two conclusions for grain refinement are consistent from EBSD and E2EM.

It is pertinent to mention that Ti element has pronounced effects on grain refinement of aluminum than Zr element, provided both are added in equal amounts. Furthermore, EBSD experiments show two kinds of crystal orientation relationships between Al/Al_3_Ti and Al/Al_3_Zr. E2EM theoretical calculation also draws the same conclusion that there exists some kind of possible crystal orientation relationship both Al/Al_3_Ti and Al/Al_3_Zr. However there also exist some contradictions between results of EBSD and E2EM. As the misfit degree of Al/Al_3_Ti in corresponding crystallography orientation has no advantage of Al/Al_3_Zr while the grain refinement efficient of Ti is superior to Zr, analysis suggests that the grain refinement of Ti in aluminum alloys has some other mechanism, which may be the effect of sufficient solutes^1,2^ and quasicrystal induced twin nucleation^35^ and is out of scope.

## Conclusions

1. Ti and Zr elements are efficient grain refiner of commercial purity aluminum and its alloys. 0.2% Ti has more tendency in grain refinement of Al than an equal amount of Zr.
2. Al_3_Ti particles in Al-Ti alloy and Al_3_Zr particles in Al-Zr alloy act as heterogeneous nucleation cores during alloy solidification.
3. The crystallography orientation relationships between Al/Al_3_Ti and Al/Al_3_Zr from both the EBSD and E2EM are as following:

The ORs of Al/Al_3_Ti:

$${\rm{OR }(1):\left\{111\right\}}_{Al}//{\left\{112\right\}}_{{Al}_{3}Ti},{\langle 110\rangle }_{Al}//{\langle 110\rangle }_{{Al}_{3}Ti}$$

$$\rm{OR }(2): {\left\{111\right\}}_{Al}//{\left\{112\right\}}_{{Al}_{3}Ti},{\langle 110\rangle }_{Al}//{\langle 021\rangle }_{{Al}_{3}Ti}$$

The ORs of Al/Al_3_Zr:

$${\rm{OR }(1):\left\{111\right\}}_{Al}//{\left\{114\right\}}_{{Al}_{3}Zr},{\langle 110\rangle }_{Al}//{\langle 110\rangle }_{{Al}_{3}Zr}$$

$${\rm{OR }(2): \left\{220\right\}}_{Al}//{\left\{220\right\}}_{{Al}_{3}Zr},{\langle 110\rangle }_{Al}//{\langle 110\rangle }_{{Al}_{3}Zr}$$
